# Surpassing genetic height potential at final adult height after monthly depot leuprolide therapy in Taiwanese girls with central precocious or early puberty: a ROC-based analysis

**DOI:** 10.3389/fped.2026.1819205

**Published:** 2026-04-28

**Authors:** Pei-Chi Lin, Yi-Wen Lai, Fu-Sung Lo

**Affiliations:** 1 Division of Pediatric Endocrinology & Genetics, Department of Pediatrics, Chang Gung Memorial Hospital, Chung Gung University College of Medicine, Taoyuan, Taiwan; 2 Department of Pediatrics, Chang Gung Memorial Hospital, Chiayi, Taiwan; 3 Department of Pediatrics, Chiayi Christian Hospital, Chiayi, Taiwan

**Keywords:** precocious puberty, GnRH agonist, final adult height, mid-parental height, bone age, growth velocity, ROC curve, treatment timing

## Abstract

**Background:**

Gonadotropin-releasing hormone agonists (GnRHa) are widely used to treat central precocious puberty (CPP), yet long-term outcomes based on confirmed final adult height (FAH) remain limited, particularly in Asian populations. Whether GnRHa therapy enables patients to surpass their genetically predicted height potential has not been well established.

**Methods:**

This retrospective cohort study included 250 girls with central precocious puberty (CPP) or early puberty treated with depot leuprolide acetate (3.75 mg every 4 weeks) at Chang Gung Memorial Hospital (2003–2023). Among 84 patients with confirmed FAH, 73 were included after excluding those receiving growth hormone. Patients were categorized as FAH > mid-parental height (MPH) or FAH ≤ MPH. Clinical and auxological variables were analyzed, and ROC analysis evaluated the predictive value of age at treatment initiation.

**Results:**

Among 73 patients, 39 (53.4%) achieved FAH exceeding MPH. These patients were younger at treatment initiation, had greater bone age advancement, and higher baseline height SDS, while absolute height was similar. During therapy, they maintained superior height SDS and growth velocity, resulting in a 6.57 cm greater FAH (*p* < 0.001). ROC analysis showed modest discrimination (AUC ∼ 0.69), and no independent predictors were identified on multivariable analysis.

**Conclusion:**

In conclusion, GnRHa therapy is associated with favorable height outcomes in a subset of patients, particularly with earlier initiation and sustained growth during treatment. These findings support a prognostic, individualized approach emphasizing skeletal maturity, pubertal tempo, and longitudinal growth monitoring.

## Introduction

Precocious puberty accelerates skeletal maturation and may lead to premature epiphyseal closure, ultimately compromising final adult height (FAH) (1–3). Gonadotropin-releasing hormone agonists (GnRHa) represent the standard therapy for central precocious puberty (CPP), effectively suppressing gonadotropin secretion, delaying pubertal progression, slowing bone age advancement, and preserving growth potential (1, 4).

Despite their established efficacy, the impact of GnRHa therapy on FAH remains variable. Height outcomes are influenced by multiple clinical and auxological factors, including chronological age at treatment initiation, degree of skeletal advancement, growth velocity during therapy, tempo of bone maturation, mid-parental height (MPH), and intrinsic growth capacity (5, 6). Although meta-analyses demonstrate an overall height benefit—particularly in girls treated before 6–8 years of age—substantial heterogeneity persists, and reliable predictors of long-term height outcome remain incompletely defined (6, 7).

Most previous studies have evaluated treatment efficacy using surrogate endpoints, such as changes in predicted adult height (PAH) or height at a predefined bone age (e.g., 14–15 years) (5–9). However, these measures may not accurately reflect true FAH and therefore limit the precision of long-term outcome assessment. Studies reporting outcomes at confirmed FAH are relatively limited, and even fewer have examined height attainment relative to genetic growth potential (7).

In recent years, increasing attention has focused on evaluating FAH in relation to MPH, a clinically accessible proxy for genetic height potential. Contemporary studies, particularly from East Asia—including cohorts from Korea, China, and Taiwan—have reported heterogeneous findings (10, 11). While some studies suggest that GnRHa therapy facilitates attainment of target height, others indicate that FAH generally approximates, rather than exceeds, MPH,particularly in patients treated at older ages or with more advanced skeletal maturation and rapidly progressive puberty. Collectively, these findings suggest that GnRHa therapy primarily preserves, rather than augments, genetic height potential, and that treatment response is strongly influenced by baseline characteristics and timing of intervention.

From a clinical perspective, MPH provides a practical reference for genetic growth expectation. Assessment of whether FAH approaches or exceeds MPH may offer additional insight beyond conventional endpoints such as PAH gain. However, exceeding MPH should be interpreted with caution, as this outcome may be influenced by baseline differences in genetic target height and statistical regression to the mean, and does not necessarily indicate a true augmentation of intrinsic growth potential. Accordingly, evaluation of FAH relative to MPH should be considered exploratory and hypothesis-generating rather than definitive evidence of treatment effect.

Furthermore, the optimal timing of GnRHa initiation remains an area of ongoing debate, particularly in girls treated after 6 years of age or those with early or slowly progressive puberty (7, 12, 13). While early treatment is generally recommended for classical CPP, the magnitude of benefit in later-onset cases is less certain. Identification of clinically useful markers to inform prognostic counseling and expectation setting is therefore of practical importance, although such markers should not be interpreted as prescriptive thresholds for treatment initiation.

In this Taiwanese cohort, we evaluated girls with CPP or early puberty who were treated with depot leuprolide acetate (3.75 mg administered subcutaneously every four weeks) and followed until attainment of confirmed FAH. We compared patients who achieved FAH above their MPH with those who did not, and explored clinical and auxological factors associated with height attainment relative to genetic expectation.

We hypothesized that earlier initiation of GnRHa therapy and less advanced skeletal maturation at treatment onset would be associated with more favorable height outcomes relative to MPH. Specifically, we explored whether bone age at treatment initiation may serve as a clinically informative, albeit modest, prognostic marker for height attainment, recognizing that its predictive performance may be limited and should be interpreted within a broader clinical context.

## Patients and methods

### Study population and definitions

This retrospective cohort study included girls diagnosed with central precocious puberty (CPP) or early puberty who were treated at the pediatric endocrinology clinics of Chang Gung Memorial Hospital (Linkou and Taipei branches) between January 2003 and December 2023. During the study period, 250 consecutive patients received gonadotropin-releasing hormone agonist (GnRHa) therapy with depot leuprolide acetate (3.75 mg administered subcutaneously every four weeks) (1, 4).

CPP was defined as the onset of secondary sexual characteristics before 8 years of age in girls, accompanied by activation of the hypothalamic–pituitary–gonadal axis, as evidenced by a pubertal response to gonadotropin-releasing hormone (GnRH) stimulation testing (peak luteinizing hormone ≥5 IU/L). Early puberty was defined as the onset of pubertal development between 8 and 10 years of age with evidence of progressive pubertal advancement, including accelerated growth velocity and/or advanced bone age.

The decision to initiate GnRHa therapy was based on a combination of clinical and biochemical factors, including age at presentation, rate of pubertal progression, degree of bone age advancement, predicted adult height (PAH) compromise, and family preference, in accordance with international clinical practice guidelines. In addition, treatment decisions were influenced by local healthcare system factors. In Taiwan, initiation and continuation of GnRHa therapy are guided by National Health Insurance reimbursement criteria in conjunction with clinician judgment, which may differ from recommendations in other countries.

Discontinuation of GnRHa therapy followed Taiwan National Health Insurance criteria, including a growth velocity of <2 cm/year or attainment of near-final growth status, defined as a bone age ≥14 years in girls (≥15 years in boys).

### Data collection and study grouping

Clinical, auxological, and treatment-related data were extracted from electronic medical records. Patients were stratified according to final adult height (FAH) relative to mid-parental height (MPH) into two groups:

- FAH > MPH
- FAH ≤ MPH

FAH was defined as the measured height at or beyond 20 years of age, or when growth velocity was <1 cm/year with radiographic evidence of epiphyseal closure.

### Auxological assessment and definitions

Height was measured using a calibrated stadiometer and recorded to the nearest 0.1 cm. Height standard deviation scores (SDS) were calculated according to World Health Organization growth references.

Bone age was assessed using the Greulich and Pyle method by an experienced pediatric endocrinologist (F.S.L.), with all assessments performed by the same evaluator to ensure consistency. Bone age advancement was defined as the difference between bone age and chronological age (BA–CA).

Predicted adult height (PAH) was estimated using the Bayley–Pinneau method, with tables selected according to the degree of skeletal maturation.

Mid-parental height (MPH) was calculated using standard sex-adjusted formulas:

$$\mathrm{MPH}\;(\mathrm{girls})=\frac{\mathrm{fathe}r^{\prime }s\;\mathrm{height}+\mathrm{mothe}r^{\prime }s\;\mathrm{height}-13}{2}$$

### Outcome definition and data collection

Final adult height (FAH) was defined as the height attained when linear growth was considered complete, as indicated by a growth velocity of <1 cm/year and/or near-complete epiphyseal maturation on bone age assessment. In this study, FAH was recorded as the measured height at or after 20 years of age, when available. This definition is consistent with established pediatric endocrinology standards (1, 12).

The following clinical and auxological variables were collected:

- Birth weight and birth length
- Chronological age and bone age at treatment initiation
- Height, body mass index (BMI), and growth velocity during treatment
- Predicted adult height (PAH) at baseline
- Duration of gonadotropin-releasing hormone agonist (GnRHa) therapy
- Menarche status prior to treatment initiation

### Follow-Up and cohort derivation

**Figure 1:**
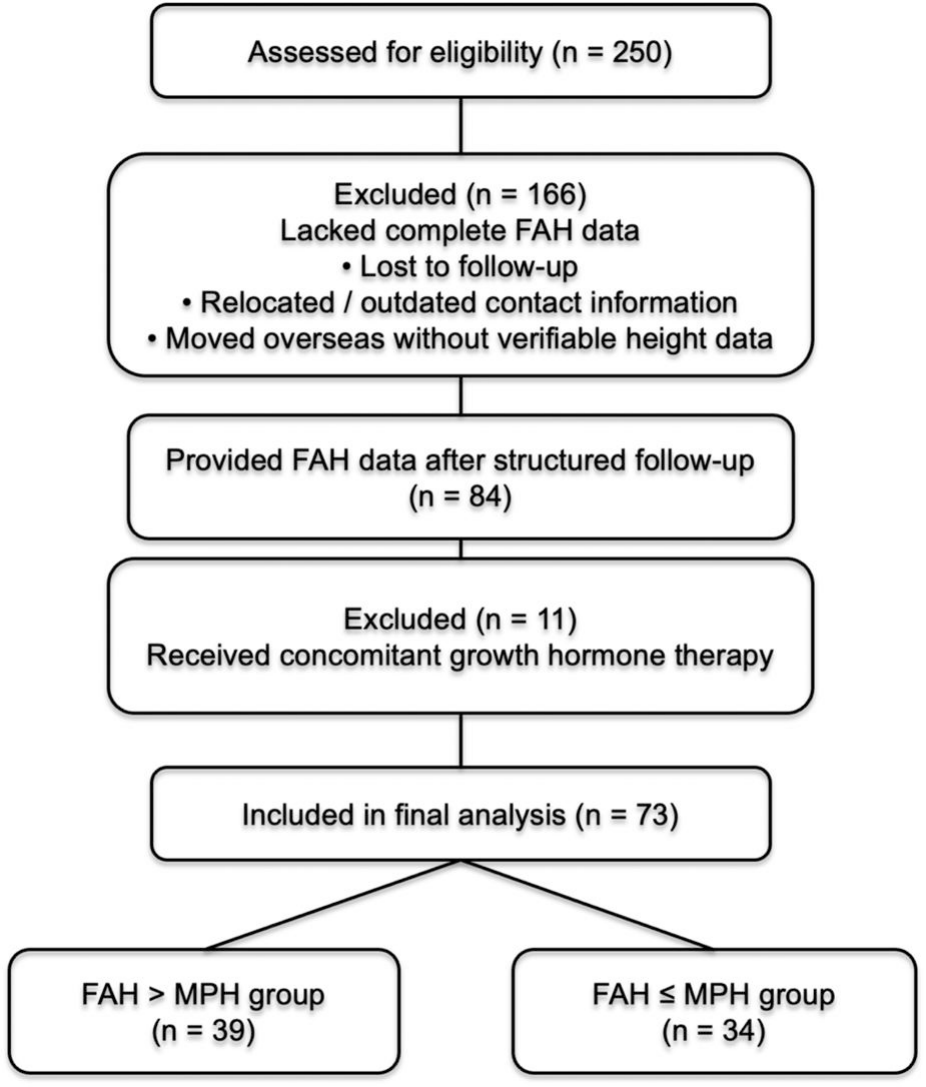
Study flow diagram in this study. Of 250 girls treated with depot leuprolide, 73 with complete adult height data and GnRHa monotherapy were included in the final analysis and categorized according to whether their final adult height exceeded mid-parental height.

Among the initial 250 patients, 166 did not have documented adult height data in the medical records. To address this, a structured follow-up protocol was implemented, including mailed consent forms and standardized telephone interviews to ascertain FAH. All contact attempts were systematically documented (Figure 1).

A total of 84 patients provided confirmed adult height data. Eleven patients who received concomitant growth hormone therapy were excluded to avoid confounding effects on growth outcomes. The final analytic cohort therefore consisted of 73 patients who received GnRHa monotherapy and achieved confirmed FAH.

Within this cohort, central precocious puberty (CPP) accounted for 45.2% (*n* = 33), and early puberty accounted for 54.8% (*n* = 40), reflecting the inclusion of patients with differing pubertal timing and progression patterns.

### Statistical analysis

All statistical analyses were performed using SAS version 9.4 (SAS Institute Inc., Cary, NC, USA).

Continuous variables are presented as mean ± standard deviation (SD), and categorical variables as frequencies and percentages. Between-group comparisons were conducted using independent-samples *t*-tests for continuous variables and chi-square or Fisher's exact tests for categorical variables, as appropriate.

Pearson correlation coefficients were calculated to evaluate associations between treatment duration, growth velocity, and height outcomes.

To identify factors associated with surpassing mid-parental height (MPH) at FAH, logistic regression analysis was performed. Variables with *P* < 0.05 in univariate analysis were entered into multivariable logistic regression models. Adjusted odds ratios (ORs) with 95% confidence intervals (CIs) were reported.

Receiver operating characteristic (ROC) curve analysis was conducted for selected clinically relevant variables, including chronological age and bone age at treatment initiation, to explore their ability to predict final adult height outcomes. The area under the curve (AUC) with corresponding 95% CIs was calculated. Optimal cutoff values were determined using the Youden index. Given the moderate discriminative performance, these analyses were considered exploratory. Model discrimination and calibration were further evaluated to assess robustness.

All statistical tests were two-sided, and a *P* value < 0.05 was considered statistically significant.

### Ethical Approval

The study was approved by the Institutional Review Board of Chang Gung Memorial Hospital (Approval No. 202401612B0). Written informed consent was obtained prior to follow-up data collection. All data were anonymized and handled in accordance with institutional and ethical standards.

## Results

### Cohort characteristics

A total of 73 girls treated with GnRHa monotherapy and followed to confirmed final adult height (FAH) were included in the analysis. Among them, 39 patients (53.4%) achieved FAH exceeding mid-parental height (MPH), whereas 34 (46.6%) did not (Table 1).

**Table 1:** Baseline clinical and auxological characteristics of girls with precocious or early puberty treated with GnRHa, stratified by whether final adult height exceeded mid-parental height.

Parameter	All patients (*n* = 73)	FAH > MPH (*n* = 39)	FAH ≤ MPH (*n* = 34)	*p*-value
	Mean ± SD	Mean ± SD	Mean ± SD	
CA, years	9.28 ± 1.30	8.84 ± 0.90	9.79 ± 1.51	0.0022
BA, years	11.65 ± 1.02	11.51 ± 0.90	11.81 ± 1.13	0.2059
BA–CA, years	2.37 ± 1.20	2.67 ± 0.99	2.02 ± 1.34	0.0201
Height, cm	135.92 ± 6.33	136.05 ± 5.96	135.78 ± 6.81	0.8595
Height SDS	0.39 ± 1.17	0.88 ± 0.89	−0.13 ± 1.23	0.0002
Weight, kg	33.78 ± 5.95	33.79 ± 5.96	33.77 ± 6.06	0.9913
Weight SDS	0.36 ± 0.96	0.61 ± 0.81	0.11 ± 1.03	0.0241
BMI, kg/m²	18.19 ± 2.25	18.17 ± 2.39	18.20 ± 2.12	0.9539
BMI SDS	0.26 ± 0.83	0.35 ± 0.84	0.16 ± 0.82	0.3211
PAH, cm	151.66 ± 4.67	152.65 ± 5.10	150.56 ± 3.91	0.058
MPH, cm	155.45 ± 3.98	154.39 ± 4.08	156.66 ± 3.55	0.0143
Birth weight, g	2,932.35 ± 458.77	2,946.25 ± 381.74	2,915.10 ± 546.23	0.7959
IGF-1, ng/mL	349.12 ± 96.07	367.00 ± 100.07	328.68 ± 99.00	0.2046
Peak LH, mIU/mL	18.41 ± 14.46	14.56 ± 10.65	25.42 ± 18.11	0.090
Peak FSH, mIU/mL	13.99 ± 6.07	13.35 ± 4.22	15.11 ± 8.66	0.579
T4, ug/dL	8.51 ± 1.49	8.68 ± 1.46	8.36 ± 1.54	0.5122
TSH, mU/L	2.57 ± 1.87	2.88 ± 2.38	2.25 ± 1.11	0.2386
Pubertal status				
Menarche^a^	20 (27.4%)	8 (20.5%)	12 (35.3%)	0.1578
Withdrawal bleeding^b^	5 (6.85%)	0 (0%)	5 (14.71%)	0.0083
Tanner stage—breast				0.4191
Stage 2	2 (2.7%)	1 (2.6%)	1 (2.9%)	
Stage 3	22 (30.1%)	15 (38.5%)	7 (20.6%)	
Stage 4	21 (28.8%)	12 (30.8%)	9 (26.5%)	
Stage 5	23 (31.5%)	10 (25.6%)	13 (38.2%)	
Tanner stage—pubic hair				0.2013
Stage 1	53 (72.6%)	32 (82.1%)	21 (61.8%)	
Stage 2	15 (20.6%)	6 (15.4%)	9 (26.5%)	
Stage 3	1 (1.4%)	0	1 (2.9%)	

FAH, final adult height; MPH, mid-parental height; GnRHa, gonadotropin-releasing hormone agonist; CA, chronological age; BA, bone age; BA–CA, bone age advancement; SDS, standard deviation score; BMI, body mass index; PAH, predicted adult height; IGF-1, insulin-like growth factor 1; LH, luteinizing hormone; FSH, follicle-stimulating hormone; T4, thyroxine; TSH, thyroid-stimulating hormone.

a Menarche indicates onset of menstruation before initiation of GnRHa therapy.

b Withdrawal bleeding indicates vaginal bleeding occurring after initiation of GnRHa therapy.

Comparisons between groups were performed using independent *t*-tests for continuous variables and chi-square or Fisher's exact tests for categorical variables, as appropriate. A *p*-value < 0.05 was considered statistically significant.

At treatment initiation, patients who ultimately surpassed MPH were significantly younger than those who did not (8.84 ± 0.90 vs. 9.79 ± 1.51 years; mean difference −0.95 years, *P* = 0.002). Chronological bone age did not differ significantly between groups; however, bone age advancement (BA–CA) was greater in the FAH > MPH group (2.67 ± 0.99 vs. 2.02 ± 1.34 years; *P* = 0.020).

Baseline absolute height was comparable between groups, but height standard deviation score (SDS) was significantly higher in the FAH > MPH group (0.88 ± 0.89 vs. −0.13 ± 1.23; mean difference 1.01 SDS units, *P* < 0.001), indicating a more favorable relative growth position at treatment initiation. Weight SDS was also modestly higher (0.61 ± 0.81 vs. 0.11 ± 1.03; *P* = 0.024), whereas BMI and BMI SDS did not differ significantly.

Notably, MPH was significantly lower in patients who ultimately exceeded their genetic target (154.39 ± 4.08 vs. 156.66 ± 3.55 cm; mean difference −2.27 cm, *P* = 0.014), suggesting lower baseline genetic height potential. Baseline predicted adult height (PAH) was higher in the FAH > MPH group but did not reach statistical significance (*P* = 0.058).

No significant differences were observed in birth parameters, IGF-1 levels, peak LH/FSH responses, or thyroid function indices. Withdrawal bleeding following GnRHa initiation occurred exclusively in the FAH ≤ MPH group (14.7% vs. 0%; *P* = 0.008), although baseline menarche status and Tanner staging were comparable.

### Longitudinal auxological response

During treatment, patients who ultimately exceeded MPH demonstrated consistently more favorable growth trajectories (Table 2).

**Table 2:** Longitudinal clinical and auxological characteristics during and after GnRHa treatment in girls with precocious or early puberty, stratified by whether final adult height exceeded mid-parental height.

Parameter	FAH > MPH	FAH ≤ MPH	*p*-value	FAH > MPH	FAH ≤ MPH	*p*-value
	After 1 year treatment			After 2 years treatment		
	(*n* = 70)			(*n* = 64)		
CA, years	9.85 ± 0.91	10.61 ± 1.34	0.009	10.87 ± 0.92	11.53 ± 1.40	0.0041
BA (years)	12.11 ± 0.66	12.40 ± 1.00	0.162	12.61 ± 0.50	12.96 ± 0.91	0.0482
BA-CA, years	2.25 ± 0.97	1.74 ± 1.23	0.0479	1.74 ± 0.83	1.33 ± 1.15	0.1006
Height, cm	141.26 ± 5.47	139.69 ± 6.44	0.2719	145.87 ± 5.6	143.7 ± 5.26	0.1250
Height SDS	0.59 ± 0.94	−0.35 ± 1.14	0.0003	0.29 ± 0.99	−0.54 ± 0.99	0.0018
Weight, kg	38.05 ± 7.09	38.13 ± 7.01	0.9624	42.97 ± 7.66	41.46 ± 7.02	0.4262
Weight SDS	0.52 ± 0.90	0.11 ± 0.93	0.0666	0.49 ± 0.88	0.03 ± 0.98	0.0506
BMI, kg/m^2^	19.01 ± 2.98	19.41 ± 2.52	0.5442	20.18 ± 3.29	19.99 ± 2.67	0.8124
BMI SDS	0.38 ± 0.94	0.33 ± 0.81	0.8481	0.50 ± 0.98	0.32 ± 0.89	0.4471
PAH, cm	154.65 ± 4.98	151.11 ± 4.76	0.0037	156.8 ± 4.97	152.45 ± 3.84	0.0004
GV (cm/year)	5.24 ± 1.03	4.42 ± 1.18	0.0034	4.57 ± 1.63	3.43 ± 1.30	0.0034
	End of treatment			Final adult height^a^		
	(*n* = 73)			(*n* = 73)		
CA, years	11.11 ± 1.63	11.56 ± 1.12	0.0584			
Duration of treatment	2.20 ± 0.85	1.75 ± 0.98	0.0390			
Height, cm	146.71 ± 4.94	143.09 ± 5.76	0.0051	158.83 ± 4.47	152.26 ± 4.50	<0.001
Height SDS	0.16 ± 1.01	−0.75 ± 1.14	0.0060	−0.07 ± 0.80	−1.25 ± 0.81	<0.001
Weight, kg	43.43 ± 7.38	41.01 ± 7.09	0.1577	55.63 ± 9.35	50.99 ± 11.54	0.0723
Weight SDS	0.40 ± 0.81	−0.08 ± 0.92	0.0214	0.30 ± 1.07	−0.23 ± 1.32	0.0719
BMI, kg/m^2^	20.14 ± 3.12	19.95 ± 2.68	0.7757	22.03 ± 3.86	22.00 ± 4.70	0.9708
BMI SDS	0.44 ± 0.90	0.28 ± 0.84	0.4497	0.35 ± 1.20	0.34 ± 1.46	0.9737
FAH-PAH at start (cm)				10.09 ± 24.99	1.70 ± 4.29	0.0455
FAH-MPH (cm)				4.43 ± 2.43	−4.44 ± 3.39	<0.001
MPH-PAH at start (cm)				5.66 ± 24.61	6.09 ± 4.61	0.9147

FAH, final adult height; MPH, mid-parental height; GnRHa, gonadotropin-releasing hormone agonist; CA, chronological age; BA, bone age; BA–CA, bone age advancement; SDS, standard deviation score; BMI, body mass index; PAH, predicted adult height; GV, growth velocity.

a Final adult height is defined as the height over 20 years old.

Comparisons between groups were performed using independent *t*-tests for continuous variables. A *p*-value < 0.05 was considered statistically significant.

In the first year, growth velocity was significantly higher in the FAH > MPH group (5.24 ± 1.03 vs. 4.42 ± 1.18 cm/year; mean difference 0.82 cm/year, *P* = 0.003), accompanied by higher height SDS (*P* < 0.001) and greater PAH (*P* = 0.004).

These differences persisted into the second year of therapy, with continued higher growth velocity (4.57 ± 1.63 vs. 3.43 ± 1.30 cm/year; *P* = 0.003), as well as sustained separation in height SDS and PAH between groups.

Treatment duration was modestly longer in the FAH > MPH group (2.20 ± 0.85 vs. 1.75 ± 0.98 years; *P* = 0.039).

At confirmed FAH, patients who exceeded MPH achieved substantially greater adult height (158.83 ± 4.47 vs. 152.26 ± 4.50 cm; mean difference 6.57 cm, *P* < 0.001). Final height SDS also differed markedly (−0.07 ± 0.80 vs. −1.25 ± 0.81; *P* < 0.001), whereas adult BMI and weight parameters were similar between groups.

### Multivariable analysis

Multivariable logistic regression analysis was performed adjusting for age at treatment initiation, baseline bone age advancement, height SDS, growth velocity, treatment duration, peak LH level, and withdrawal bleeding (Table 3).

**Table 3:** Multivariable logistic regression analysis for predictors of exceeding mid-parental height (FAH > MPH).

Variable	Odds Ratio (OR)	95% Confidence Interval	*p*-value
Age at GnRHa initiation, years	0.754	0.008–69.567	0.903
Height SDS after 1 year of treatment	0.218	0.021–2.295	0.205
Growth velocity during year 1, cm/year	3.884	0.393–38.412	0.246
Growth velocity during year 2, cm/year	0.324	0.057–1.840	0.204
Withdrawal bleeding (yes vs. no)	4.279	0.028–664.772	0.963
Duration of GnRHa treatment, years	1.928	0.110–33.741	0.653
Peak LH, IU/L	1.035	0.947–1.131	0.444
Baseline bone age advancement (BA–CA), years	1.993	0.375–10.589	0.418

Multivariable logistic regression analysis was performed to identify independent predictors associated with exceeding mid-parental height (FAH > MPH) among girls treated with GnRHa. Variables included demographic, auxological, and treatment-related factors selected based on clinical relevance and univariate analysis. Odds ratios (OR) represent the change in odds of exceeding MPH per unit increase in each predictor variable. Confidence intervals (CI) represent 95% confidence intervals. A *p*-value < 0.05 was considered statistically significant.

No variable emerged as an independent predictor of surpassing MPH. Confidence intervals were wide across several covariates, suggesting limited statistical power. In addition, potential collinearity among growth-related variables (e.g., age, height SDS, and growth velocity) may have attenuated independent associations.

These findings indicate that exceeding genetic height potential is likely determined by multifactorial growth dynamics rather than a single dominant predictor.

### Predictive performance of treatment timing

**Figure 2:**
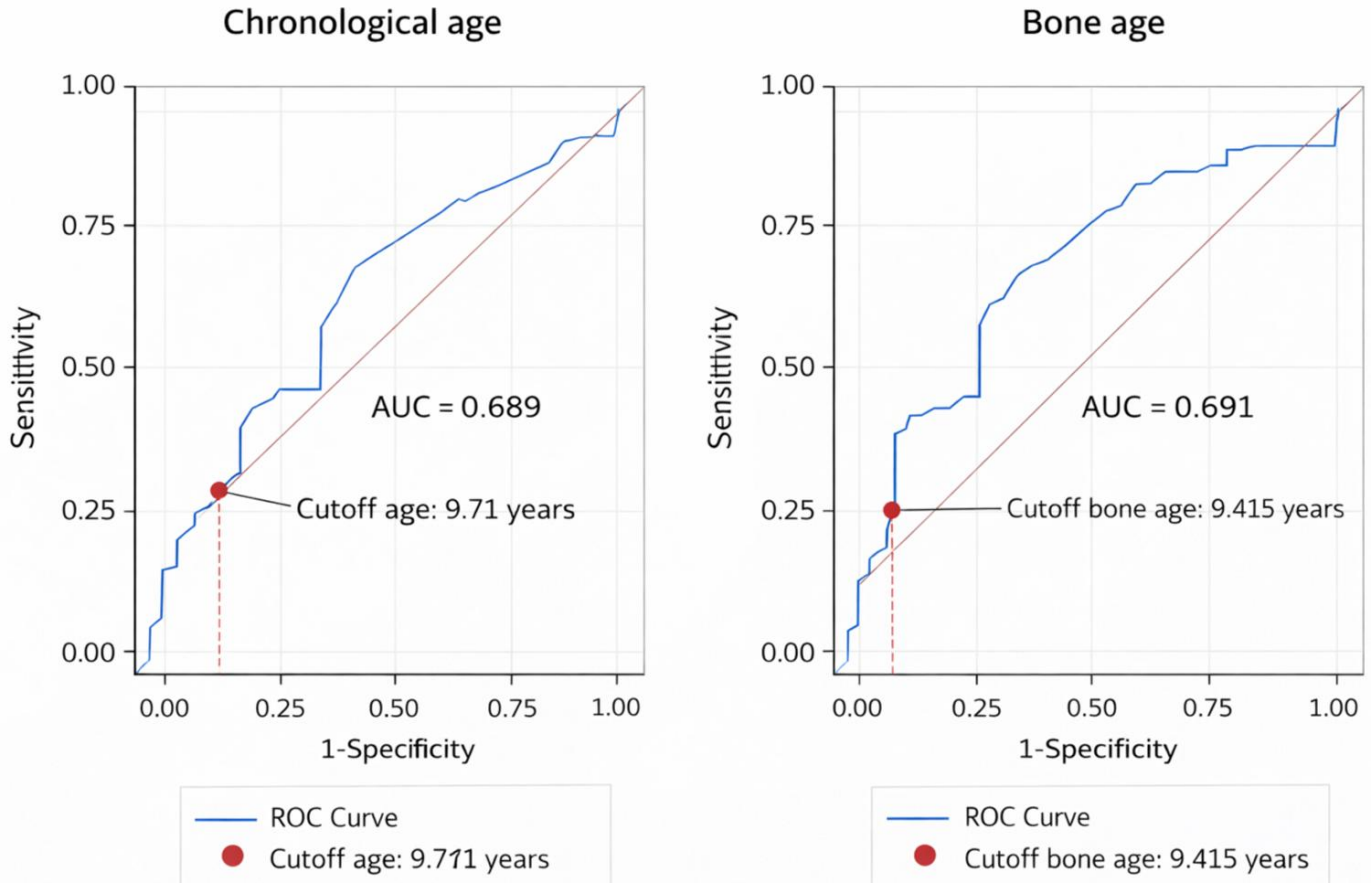
Receiver operating characteristic (ROC) curves of chronological age and baseline bone age at GnRHa initiation for predicting exceeding mid-parental height. Receiver operating characteristic (ROC) curves demonstrating the predictive performance of chronological age (left panel) and baseline bone age (right panel) at initiation of gonadotropin-releasing hormone agonist (GnRHa) therapy for achieving final adult height exceeding mid-parental height (FAH > MPH). ROC, receiver operating characteristic; AUC, area under the curve; GnRHa, gonadotropin-releasing hormone agonist; FAH, final adult height; MPH, mid-parental height.

Receiver operating characteristic (ROC) analysis was conducted to evaluate the discriminative ability of chronological age and bone age at treatment initiation for predicting FAH > MPH (Figure 2).

Chronological age demonstrated modest discriminative performance (AUC = 0.689), with an optimal cutoff of 9.71 years based on the Youden index. Baseline bone age showed a similar level of discrimination (AUC = 0.691), with an optimal cutoff of 9.415 years.

Although bone age demonstrated slightly higher discriminative performance compared with chronological age, both models showed only moderate predictive ability. Therefore, these thresholds should be interpreted as exploratory and hypothesis-generating rather than definitive clinical decision tools.

## Discussion

In this retrospective cohort study, we evaluated long-term height outcomes in girls with central precocious puberty (CPP) or early puberty treated with gonadotropin-releasing hormone agonist (GnRHa), using confirmed final adult height (FAH) in relation to mid-parental height (MPH) as the principal outcome. Among the 73 patients with available FAH data, 39 (53.4%) achieved adult height exceeding MPH (6, 8, 12, 14). However, this finding should be interpreted cautiously, as the analyzed cohort represented only a subset of the original treated population, and several methodological and statistical considerations limit causal inference.

A major strength of the study is the use of confirmed FAH rather than predicted adult height (PAH). Although PAH is widely used in studies of pubertal suppression (5–9), it remains an indirect estimate that is influenced by assumptions regarding skeletal maturation and prediction models. By focusing on achieved adult stature, our study provides a more clinically meaningful assessment of long-term growth outcome after GnRHa treatment (1, 7, 14, 15).

Patients who ultimately achieved FAH > MPH were significantly younger at treatment initiation and had greater bone age advancement at baseline, together with more favorable height SDS and longitudinal growth patterns during therapy. These findings suggest that treatment initiated at an earlier stage of pubertal and skeletal maturation may be associated with better height outcomes (1, 4, 6, 8, 12, 14). ROC analysis further showed that both chronological age and bone age at treatment initiation had modest discriminative ability, with AUC values of approximately 0.69. These results indicate that earlier treatment initiation is associated with a higher probability of favorable height outcome, but the predictive performance is only moderate. Accordingly, the identified cutoffs should not be interpreted as definitive treatment thresholds, but rather as exploratory values that may help inform counseling and expectation setting.

### Timing of intervention: skeletal maturity as a biological framework

Greater bone age advancement (BA–CA) at treatment initiation was associated with achieving FAH exceeding MPH, likely reflecting pubertal tempo rather than skeletal maturity alone. Patients with more advanced BA may represent rapidly progressive CPP, in whom early growth acceleration combined with timely GnRHa suppression preserves residual growth potential, whereas those with lesser BA advancement may include slowly progressive or early puberty cases with more limited treatment benefit.

Bone age more directly reflects skeletal maturation and remaining growth potential than chronological age; however, the modest predictive performance of both variables underscores that adult height outcome is multifactorial and not determined by age alone. In clinical practice, treatment timing should therefore be guided by an integrated assessment of pubertal progression, bone age advancement, growth velocity, and predicted height compromise.

Notably, treatment benefit appears to follow a graded relationship with skeletal maturity rather than a strict age cutoff. Even beyond the traditional window (<6–8 years) (6, 12, 13, 16–18), a maturation-dependent opportunity for intervention may persist, supporting a biologically informed approach to clinical decision-making (1, 19).

### Longitudinal growth dynamics, genetic context, and treatment responsiveness

Patients who ultimately exceeded mid-parental height (MPH) demonstrated sustained advantages in height SDS and growth velocity throughout therapy, particularly during the second treatment year. This pattern suggests preserved growth plate responsiveness under effective gonadotropin suppression. Importantly, longitudinal growth trajectories—rather than baseline characteristics alone (20)—appear to provide meaningful insight into treatment responsiveness. Height SDS progression and growth velocity likely integrate intrinsic growth potential, adequacy of hormonal suppression, and overall physiological status, supporting the role of dynamic monitoring in individualized treatment optimization.

Notably, withdrawal bleeding was observed exclusively in patients who did not exceed MPH. Although infrequent, this finding may reflect incomplete suppression or altered pubertal dynamics that could compromise residual growth potential (2).

An important contextual finding was that patients who exceeded MPH had significantly lower baseline MPH. This observation warrants cautious interpretation. While it is possible that individuals with lower genetic targets derive proportionally greater benefit from timely pubertal suppression (8, 12, 14), the finding may also reflect regression toward the mean and baseline asymmetry, whereby surpassing a lower target is inherently more likely. Accordingly, exceeding MPH should not be interpreted as evidence that GnRHa therapy enables patients to surpass their genetic growth potential, but rather as an exploratory outcome influenced in part by baseline differences. Nevertheless, the approximately 6 cm difference in final adult height between groups suggests a clinically meaningful magnitude of effect (1).

Multivariable logistic regression did not identify independent predictors of exceeding MPH. However, this likely reflects the interdependence of growth-related variables and limited statistical power rather than absence of biological association. Chronological age, bone age advancement, height SDS, and growth velocity represent overlapping dimensions of the same maturation axis (6). Given that growth regulation operates as a complex and dynamic system, isolating a single dominant predictor may oversimplify the underlying biology (20). The wide confidence intervals further underscore the constraints imposed by sample size in long-term final adult height studies (11).

### Strengths and limitations

This study has several strengths. It employed a uniform GnRHa treatment protocol (1, 4), minimized interobserver variability through bone age assessment by a single experienced evaluator, and utilized confirmed final adult height (FAH) rather than surrogate endpoints (8, 11). In addition, the study provides long-term outcome data from an East Asian population, contributing to a relatively underrepresented body of literature.

Nevertheless, important limitations should be considered. First, only 73 of the initial 250 patients were included in the final analysis, introducing potential selection bias and limiting generalizability. Second, treatment discontinuation in Taiwan is guided by National Health Insurance criteria based on near-final growth (e.g., bone age ≥14 years in girls), which occurs later than international recommendations and may attenuate the potential height benefit of therapy. Third, the inclusion of both central precocious puberty and early puberty introduces clinical heterogeneity that may confound outcome interpretation.

Fourth, the definition of FAH exceeding mid-parental height (MPH) warrants cautious interpretation. The lower baseline MPH observed in the FAH > MPH group raises the possibility of regression to the mean, potentially inflating the apparent treatment effect. Fifth, the ROC analysis was restricted to chronological age and bone age; the modest discriminative performance (AUC ≈ 0.69) indicates limited predictive utility and should not be overinterpreted. Furthermore, the multivariable model may be susceptible to overfitting given the sample size and number of covariates included. Finally, residual confounding from unmeasured variables cannot be excluded (2, 11).

Overall, these findings should be interpreted within a hypothesis-generating and prognostic framework rather than as definitive evidence for causal treatment effects.

### Clinical implications

These findings support the importance of timely clinical evaluation and intervention before advanced skeletal maturation, rather than reliance on fixed chronological age thresholds (1, 12). Earlier initiation of GnRHa therapy—particularly at lower levels of bone age—was associated with more favorable height outcomes. However, the proposed bone age threshold (approximately 9.4 years) should be interpreted cautiously, given the modest discriminatory performance observed, and is best applied for prognostic counseling rather than prescriptive decision-making.

In addition to baseline characteristics, longitudinal growth patterns during therapy appear to provide meaningful clinical insight. Sustained advantages in growth velocity and height SDS were associated with improved adult height outcomes, suggesting that dynamic treatment responsiveness may better reflect intrinsic growth potential and adequacy of pubertal suppression than baseline predictors alone (20, 21). These findings underscore the importance of ongoing growth monitoring as a key component of individualized management.

From a clinical perspective, reframing treatment success as attainment of adult height relative to mid-parental height (MPH) may offer a more patient-centered and clinically meaningful framework for shared decision-making (8, 14). However, exceeding MPH should not be interpreted as definitive evidence of treatment-induced augmentation of genetic potential, as this outcome may be influenced by baseline differences in target height and regression to the mean.

Overall, this study demonstrates that GnRHa therapy is associated with favorable adult height outcomes in girls with central precocious puberty or early puberty, particularly when initiated at earlier stages of skeletal maturation (8, 12, 14). Nevertheless, the observation that a substantial proportion of patients exceeded their genetic target height should be interpreted as a relative indicator of growth trajectory modification, shaped by both biological and statistical factors.

Future prospective, multicenter studies incorporating genetic, environmental, and longitudinal growth parameters are warranted to refine prediction models, minimize bias, and optimize individualized treatment strategies for long-term growth outcomes.

## Data Availability

The original contributions presented in the study are included in the article/Supplementary Material, further inquiries can be directed to the corresponding author.
